# Lovastatin Inhibits VEGFR and AKT Activation: Synergistic Cytotoxicity in Combination with VEGFR Inhibitors

**DOI:** 10.1371/journal.pone.0012563

**Published:** 2010-09-03

**Authors:** Tong T. Zhao, Diane Trinh, Christina L. Addison, Jim Dimitroulakos

**Affiliations:** 1 Centre for Cancer Therapeutics, the Ottawa Hospital Research Institute, Ottawa, Ontario, Canada; 2 The Faculty of Medicine and the Department of Biochemistry at the University of Ottawa, Ottawa, Ontario, Canada; Universidade Federal do Rio de Janeiro, Brazil

## Abstract

**Background:**

In a recent study, we demonstrated the ability of lovastatin, a potent inhibitor of mevalonate synthesis, to inhibit the function of the epidermal growth factor receptor (EGFR). Lovastatin attenuated ligand-induced receptor activation and downstream signaling through the PI3K/AKT pathway. Combining lovastatin with gefitinib, a potent EGFR inhibitor, induced synergistic cytotoxicity in a variety of tumor derived cell lines. The vascular endothelial growth factor receptor (VEGFR) and EGFR share similar activation, internalization and downstream signaling characteristics.

**Methodology/Principal Findings:**

The VEGFRs, particularly VEGFR-2 (KDR, Flt-1), play important roles in regulating tumor angiogenesis by promoting endothelial cell proliferation, survival and migration. Certain tumors, such as malignant mesothelioma (MM), also express both the VEGF ligand and VEGFRs that act in an autocrine loop to directly stimulate tumor cell growth and survival. In this study, we have shown that lovastatin inhibits ligand-induced VEGFR-2 activation through inhibition of receptor internalization and also inhibits VEGF activation of AKT in human umbilical vein endothelial cells (HUVEC) and H28 MM cells employing immunofluorescence and Western blotting. Combinations of lovastatin and a VEGFR-2 inhibitor showed more robust AKT inhibition than either agent alone in the H28 MM cell line. Furthermore, combining 5 µM lovastatin treatment, a therapeutically relevant dose, with two different VEGFR-2 inhibitors in HUVEC and the H28 and H2052 mesothelioma derived cell lines demonstrated synergistic cytotoxicity as demonstrated by MTT cell viability and flow cytometric analyses.

**Conclusions/Significance:**

These results highlight a novel mechanism by which lovastatin can regulate VEGFR-2 function and a potential therapeutic approach for MM through combining statins with VEGFR-2 inhibitors.

## Introduction

Angiogenesis is an important physiological process during fetal development and growth as well as in mature tissue remodeling and repair [1]. For cancer expansion and dissemination, both primary lesions and metastatic tumors must develop a new vascular supply in order to survive [1]. Angiogenesis is tightly regulated by balancing the activity of pro- and anti-angiogenic factors [2]. Multiple pathways contribute to tumor angiogenesis including vascular endothelial growth factor (VEGF), fibroblast growth factor, and platelet-derived growth factor [2]. Based on the central role of VEGF in tumor angiogenesis and growth, it has emerged as a promising therapeutic target for angiogenesis inhibition [3]. VEGF, a 35- to 45-kDa dimeric polypeptide, plays a critical role in normal and pathologic angiogenesis [3]. The VEGF family includes VEGF-A, VEGF-B, VEGF-C, VEGF-D, VEGF-E, and placental growth factors 1 and 2 [4]. The VEGF-A gene, via alternative splicing, yields several isoforms, of which, VEGF_165_ plays a critical role in tumor angiogenesis [3]. Tumor cells secrete VEGF in response to many stimuli including hypoxia, low pH, or cellular stress, which are prevalent in most solid tumors [5].

VEGF exerts its biologic effect through interaction with receptors present on the cell surface. These receptor tyrosine kinases (RTK) include VEGFR-1 (Flt-1) and VEGFR-2 (KDR, Flk-1), which are predominantly present on vascular endothelial cells [6]. Both VEGFR-1 and VEGFR-2 have an extracellular ligand binding domain, a transmembrane region, and a tyrosine kinase domain [2], [3]. In addition, VEGFR-3 (Flt-4) is expressed on vascular and lymphatic endothelium while the neuropilin receptor is expressed on vascular endothelium and neurons [2], [3]. VEGFR-2 is the main receptor responsible for mediating the proangiogenic effects of VEGF in tumor-associated endothelium [7]. VEGF binding to the extracellular domain of the VEGFR results in dimerization and autophosphorylation of the intracellular tyrosine kinases [8]. This activates multiple downstream proteins that play functional roles in cell survival, proliferation vascular permeability and stabilization of new blood vessels [8]. For example, VEGF induces endothelial cell proliferation by activating the protein kinase Ras-MEK-ERK pathway [8]. The pro-survival effects of VEGF/VEGFR-2 are mediated by the PI3K/AKT pathway [8]. Recent studies indicate that VEGFR are also expressed by some tumor cells and may represent an additional target [9].

Malignant mesothelioma (MM) is a highly aggressive tumor that arises from the surface serosal cells of the pleura and, less frequently, the peritoneum [10]. A strong link has been established between exposure to asbestos and increased risk for MM [11]. Treatment of MM with surgery, chemotherapy, or radiation therapy is rarely curative and median survival is in the range of 10–17 months [11]. Novel therapies for MM are needed. VEGF up-regulation appears to play an important role in mesothelial cell transformation. High levels of VEGF have been observed in the serum of MM patients and elevated pleural effusion VEGF levels are associated with poor survival in patients with MM [12]. VEGF may also act in a functional autocrine loop capable of directly stimulating the growth of MM cells [9]. MM cell lines express elevated levels of both VEGF and the VEGFR-1 and 2 compared with normal mesothelial cells [9]. VEGF activated these receptors and increased proliferation of all MM cell lines examined [9]. Interestingly, significant vascularization is rarely exhibited in MM suggesting that VEGF may play a key role in MM tumor progression by primarily regulating tumor cell proliferation suggesting VEGF/VEGFR as therapeutic targets in MM [10].

The rate-limiting step of the mevalonate pathway is the conversion of HMG-CoA to mevalonate, which is catalyzed by HMG-CoA reductase [13]. The mevalonate pathway produces various end products that are critical for many different cellular functions including cholesterol, dolichol, ubiquinone, isopentenyladenine, geranylgeranyl pyrophosphate (GGPP), and farnesyl pyrophosphate (FPP) [13]. Geranylgeranyl transferase and farnesyl transferase use GGPP and FPP, respectively, for post-translational modifications of a wide variety of cellular proteins including the Ras, Rab, and Rho families [14], [15]. These proteins regulate cell proliferation, intracellular trafficking and cell motility and this post-translational modification functions as a membrane anchor critical for their activity [14], [15]. Blockade of the rate-limiting step of the mevalonate pathway by HMG-CoA reductase inhibitors results in decreased levels of mevalonate and its downstream products [16] and, thus, may have significant influences on many critical cellular functions.

Malignant cells appear highly dependent on the sustained availability of the end products of the mevalonate pathway [17]. The statin family of drugs are potent inhibitors of HMG-CoA reductase that are widely used as hypercholesterolemia treatments [16]. Mevalonate metabolites are required for the proper function and localization of a number of downstream mediators of the VEGFR-2 signaling cascade [3], [18], [19], [20]. Proteins that require FPP or GGPP posttranslational modifications play critical roles in transducing these signals [3], [18], [19], [20]. In our recent studies, we have demonstrated that lovastatin treatment inhibits ligand-induced activation of EGFR [18], [21]. The mechanism by which EGFR inhibition is mediated by lovastatin is novel and suggests a previously unrecognized process controlling EGFR activity.

Due to the potential of lovastatin to target EGFR function and its downstream signaling, we previously evaluated the effects of combining lovastatin with the clinically relevant EGFR tyrosine kinase inhibitor (TKI) gefitinib [22]. The combination of gefitinib and lovastatin demonstrated significant co-operative cytotoxic effects when cells were pretreated with lovastatin for 24 hrs. At this time point, lovastatin demonstrated significant inhibition of EGFR function [21]. We demonstrated co-operative cytotoxic effects with this combination that was synergistic due to the induction of a potent apoptotic response [21]. In this study, we evaluated the potential of lovastatin to similarly inhibit VEGFR-2 function. Furthermore, we evaluated the effects of lovastatin on endothelial cell proliferation and survival as well as the effects of combining lovastatin with VEGFR-TKIs on MM tumor cell viability as a potential novel therapeutic approach.

## Results

### Lovastatin inhibits internalization and degradation of the VEGFR-2

**Figure 1:**
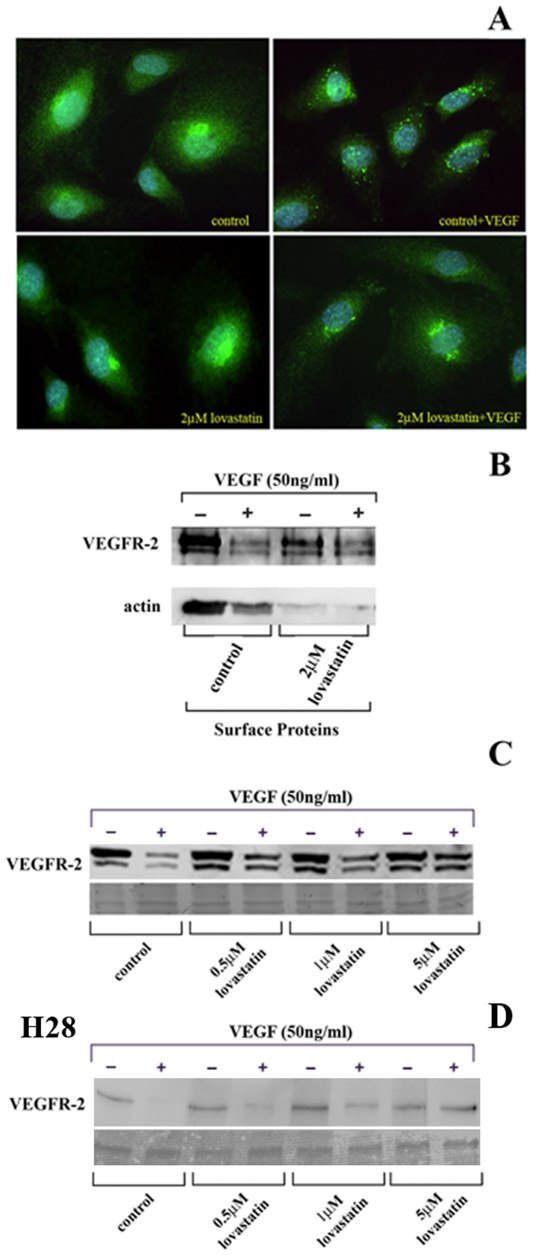
Lovastatin treatment inhibits VEGFR-2 internalization. A, VEGFR-2 internalization in HUVEC was evaluated by immunofluorescence. HUVEC were treated with solvent control or 2 µM lovastatin for 24 hrs in serum-free media followed by 30 min of stimulation with VEGF_165_. Immunofluorescence staining of HUVEC revealed a punctate intracellular staining pattern upon VEGF_165_ ligand binding in the control but not in cells treated with 2 µM lovastatin. The data is typical of 3 independent experiments. B, Cell Surface Pinpoint Protein Isolation revealed a decrease in VEGFR-2 on the surface of control HUVEC upon VEGF stimulation but not with 2 µM lovastatin treatment. Actin was readily pulled down in control cells but not in lovastatin treated HUVEC indicating a lack of association of surface proteins with actin in lovastatin treated cells. C and D, Western blot analysis reveals that VEGFR-2 receptor levels decrease with 30 min of stimulation with VEGF_165_ stimulation in control HUVEC and H28 cells respectively. Lovastatin treatments of 0.5, 1 and 5 µM inhibited VEGFR degradation in a dose dependant manner. The data is typical of at least 3 independent experiments and the membranes were stained with Ponceau Red to visualize total protein loading.

Previous studies have demonstrated that ligand binding to VEGFR-2 leads to receptor dimerization and autophosphorylation [8]. Autophosphorylation leads to the activation of its downstream signaling cascades and receptor internalization and degradation in lysosomes [8]. In this study, we evaluated the effect of lovastatin on VEGFR-2 internalization and degradation in VEGF treated HUVEC cells. Localization of VEGFR-2 was visualized by immunofluorescence staining. HUVEC cells were exposed to solvent control with or without treatment of 50 ng/ml VEGF_165_ for 30 min. In un-stimulated HUVEC cells, VEGFR-2 showed a dispersed staining pattern on the cell surface. With the addition of VEGF_165_, however, VEGFR-2 showed a distinct punctate intracellular staining pattern indicating efficient internalization of this receptor [23] in HUVEC (Figure 1A). Treatment of HUVEC with 2 µM lovastatin for 24 hrs showed a similar diffuse surface-staining pattern for VEGFR-2 as control cells. Addition of 50 ng/ml of VEGF_165_ for 30 min in lovastatin treated cells significantly reduced the punctuate intracellular staining pattern shown in control VEGF_165_ treated cells but displayed a similar diffuse staining pattern to control un-stimulated cells (Figure 1A).

To further examine whether lovastatin is regulating the internalization of the VEGFR ligand complex, we performed the Pinpoint Cell Surface Protein Isolation method that specifically labels and isolates proteins found on the cell surface. Cell surface proteins were biotinylated and isolated using immobilized avidin, prior to Western blotting with the VEGFR-2 antibody. As shown in Figure 1B, untreated HUVEC were found to have significant levels of VEGFR-2 expressed on the cell surface. As expected, stimulation with VEGF_165_ at 50 ng/ml for 30 min decreased the levels of VEGFR-2 on the cell surface (Figure 1B). In 2 µM lovastatin treated cells for 24 hrs, lower levels of surface expression of VEGFR were evident. This decrease may be the result of the inhibition of intracellular transport that is regulated in part by the geranylgeranylated rab protein family. Ligand stimulation did not affect VEGFR-2 surface expression in lovastatin treated cells indicative of inhibition of internalization. In untreated cells, actin was readily detected in the avidin pull downs while lovastatin treated cells had significantly lower levels (Figure 1B). These results suggest that in lovastatin treated HUVEC; surface protein binding of actin was inhibited. These results correspond well with recent studies that demonstrate a role for the actin cytoskeleton in the multi-step process of receptor internalization [24], [25].

Internalization of ligand bound VEGFR-2 often leads to its degradation in lysosomes as a way to attenuate its signal. To determine the effect of lovastatin on VEGFR-2 degradation, we performed Western blot analyses of total cellular protein extracted from VEGF_165_ stimulated HUVEC and H28 MM cells with or without lovastatin treatments. In HUVEC, the basal levels of VEGFR-2 were unchanged with or without 0.5, 1 and 5 µM lovastatin treatments for 24 hrs (Figures 1C and D). Control HUVEC cells stimulated with 50 ng/ml VEGF_165_ for 30 min demonstrated a significant decrease in VEGFR-2 protein levels indicating efficient degradation of ligand bound VEGFR-2 in these cells (Figures 1C and D). Treatment of HUVEC with 0.5, 1 and 5 µM lovastatin for 24 hrs attenuated the effect of VEGF_165_ addition on VEGFR-2 degradation as the levels of VEGFR-2 were significantly elevated in lovastatin-treated in comparison to control cells (Figures 1C and D). Ponceau Red staining of the membranes confirmed equal loading between samples and the area of the blot shown corresponds to the area where VEGFR-2 migrated. These results indicate that lovastatin treatment inhibits ligand-induced internalization and degradation of VEGFR-2 in HUVEC and H28 MM cells.

**Figure 2:**
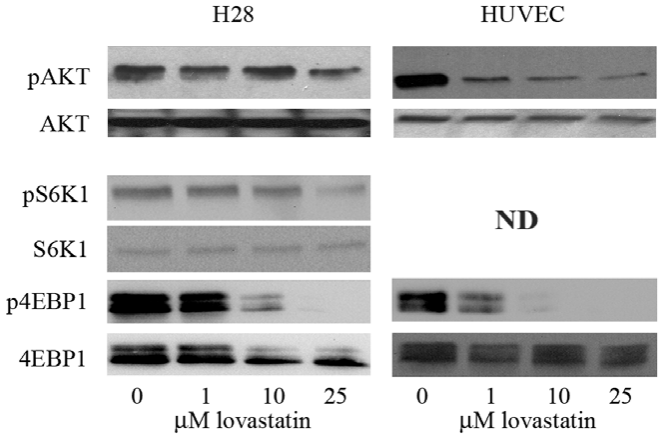
Lovastatin inhibits VEGF induced activation of AKT and its downstream targets. Cell lysates from HUVEC and H28 cells were collected following control, 1, 10 and 25 µM 24 hr lovastatin treatments in serum-free media with 50 ng/ml 30 min VEGF_165_ stimulation. Phosphorylation level of AKT decreased with lovastatin treatment in a dose dependent manner. Expression level of total AKT was assayed as the loading control. Phosphorylation levels of S6K1 and 4EBP1 also decreased with lovastatin treatment in a dose dependent manner. Phosphorylated S6K1 in HUVEC cells was not detectable (ND). Expression levels of total S6K1 and 4EBP1 were assayed as the loading control.

Based on lovastatin's ability to inhibit ligand-induced internalization of VEGFR-2, we further evaluated the effect of lovastatin treatment on the signaling cascades triggered by VEGFR-2 activation. The PI3K/AKT signaling pathway plays a significant role in cell survival responses mediated by VEGFR-2 [3]. Ligand bound VEGFR-2 activates PI3K that phosphorylates the phospholipid PIP2 resulting in the accumulation of PIP3 that in turn activates AKT [26]. Serum starved H28 MM derived cell line and HUVEC cells were treated with 0, 1, 10 and 25 µM lovastatin for 24 hrs followed with 50 ng/ml VEGF_165_ stimulation for 30 min. The functional activation of this pathway was evaluated by Western blot analysis, employing phospho-specific antibody recognizing the active form and control antibody for total AKT. Lovastatin treatment inhibited activation of AKT in a dose dependent manner that was readily detectable at the 1 µM dose in HUVEC but was less efficient in inhibiting AKT activation in H28 cells (Figure 2). There are a wide variety of AKT targets that regulate its effects on protein translation, proliferation and cell survival. These targets include ribosomal S6 kinase (S6K1) and eukaryotic translation initiation factor 4E (eIF4E) that regulate translation [27]. We evaluated the effects of lovastatin on ligand-induced activation of these proteins in our 2 model cell lines. Western blot analysis determined the effects of 0, 1, 10 and 25 µM lovastatin treatment for 24 hrs with 30 min 50 ng/ml VEGF addition on these AKT targets. Lovastatin treatment significantly inhibited phosphorylation of S6K1 (not detected in HUVEC) and 4EBP1 in a dose dependent manner (Figure 2). Activated phosphorylated AKT, S6K1 and 4EBP1 were not detected in serum starved control cells (data not shown). These results demonstrate the ability of lovastatin to readily inhibit VEGF induced AKT activation in these cell lines.

### Lovastatin induces cytotoxicity of HUVEC and MM Cells

**Figure 3:**
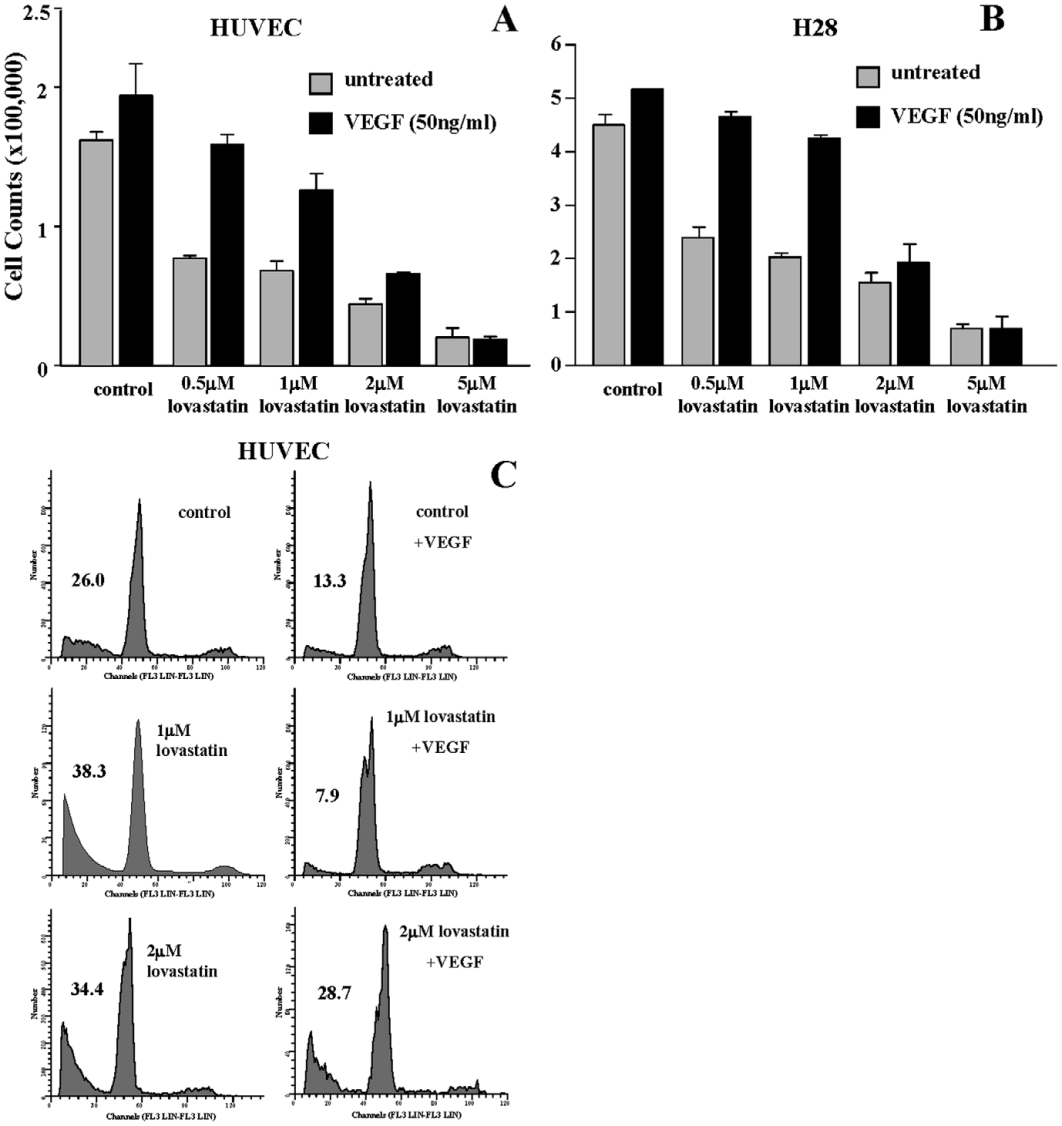
VEGF can partially rescue the cytotoxic and apoptotic effects of lovastatin. A and B, HUVEC and H28 cell proliferation was measured with a cell viability assay following either control or 0.5–5 µM 72 hr lovastatin treatments with or without 50 ng/ml VEGF_165_. VEGF_165_ stimulated control cells to proliferate, however, higher doses of lovastatin inhibited the proliferative effects of VEGF_165_. The data were normalized to untreated (media alone) cells (representing 100%) and are representative of 4 independent experiments. C, Apoptosis was measured using flow cytometric analysis of HUVEC following either control or 1 and 2 µM 72 hr lovastatin treatments with or without 50 ng/ml VEGF_165_. Results demonstrated that lovastatin was preventing the apoptotic inhibitory effects of VEGF_165_ at higher doses (2 µM). The data is typical of 2 independent experiments.

Due to the regulation of cell viability by the AKT pathway, we evaluated the effects of lovastatin treatment on HUVEC and H28 cell viability. Cell viability assays based on trypan blue exclusion cell counts of HUVEC and H28 cells were evaluated at 72 hrs. The effect on cell viability of exogenous addition of VEGF_165_ was included in this study to determine the role of this pathway in regulating lovastatin-induced cytotoxicity. Treatment with lovastatin alone at 0.5, 1, 2 and 5 µM concentrations resulted in a dose-dependant decrease in the percentage of viable cells (Figures 3A and B). VEGF_165_ proliferative effects were observed in control cells (Figures 3A and B). The addition of VEGF_165_ to lovastatin treated cells inhibited lovastatin induced cytotoxicity at the low 0.5 and 1 µM lovastatin doses but this compensatory effect was reduced or eliminated at the higher 2 and 5 µM lovastatin treated cells (Figures 3A and B). The percentage of apoptotic HUVEC 72 hrs (Figure 3B) post-treatment was assessed using propidium iodide flow cytometry to study the effects of lovastatin in inducing apoptosis. The control cells showed a sub-G1 peak in the DNA histogram that is characteristic of apoptotic cells representing approximately 26% of cells analyzed, while addition of VEGF_165_ resulted in a reduction of apoptotic cells to approximately 13%, highlighting the role of VEGF in promoting HUVEC cell survival. At a dose of 1 µM and 2 µM, lovastatin induced significant apoptosis above the levels of that observed in the control cells. However, for the 1 µM lovastatin concentration, VEGF_165_ was still able to able to diminish the apoptotic effects of lovastatin on HUVEC but with the higher 2 µM lovastatin dose, addition of VEGF_165_ had no significant affect on the induction of apoptosis (Figure 3B). The cell viability and flow cytometric analyses show the ability of lovastatin to induce a potent apoptotic response in HUVEC that at lower doses can be rescued by VEGF but not at the higher doses relevant for use of lovastatin as an anti-cancer therapeutic [28], [29].

### Lovastatin affects cytoskeleton organization and RhoA Activity

**Figure 4:**
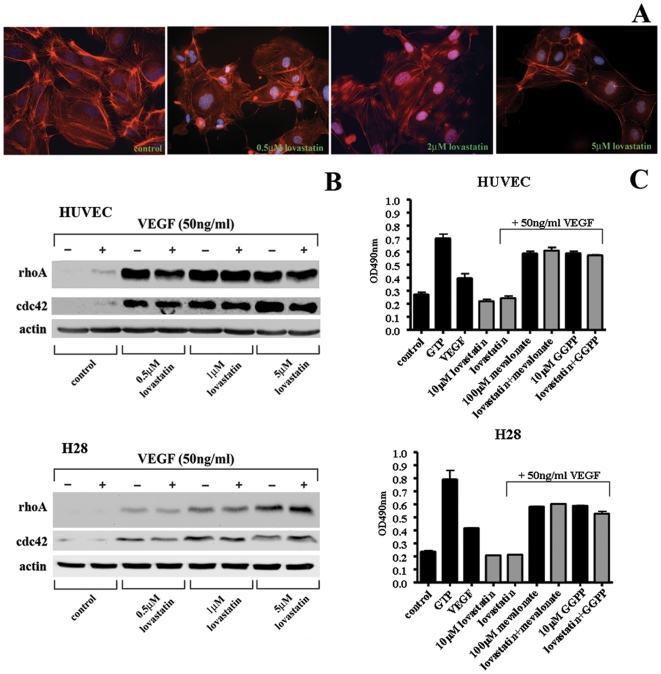
Lovastatin treatment results in actin disorganization and inhibits VEGF induced rhoA activation. A, Actin cytoskeletal organization was visualized using rhodamine-conjugated phalloidin following 24 hr 0.5, 2 and 5 µM lovastatin treatments of HUVEC. Staining revealed a lovastatin induced decrease in F-actin fibers along with a disorganized pattern. The data is typical of 3 independent experiments. B, Western blot analysis of various downstream targets of the VEGF receptor in HUVEC and H28 cells. Cell lysates were collected following 24 hr lovastatin treatment in serum-free media and either control or 30 min VEGF_165_ stimulation. Total levels of RhoA and Cdc42 increase with increasing concentrations of lovastatin irrespective of VEGF_165_ stimulation. Total levels of cyclin D1 drop as the concentration of lovastatin is increased. C, Rho A activation assays. Serum starved HUVEC and H28 cells were treated with 10 µM lovastatin, 100 µM mevalonate and 10 µM GGPP alone and in combination as indicated for 24 hrs. Cells were stimulated with VEGF for 30 min as indicated and assayed for rhoA activity employing the RhoA G-LISA kit that quantifies activated GTP loaded rhoA through colorimetric detection of rhoA bound to Rho-GTP-binding protein.

Actin cytoskeletal organization is known to play a significant role in the internalization and intracellular trafficking of RTK including VEGFRs. RhoA and cdc42 regulate actin cytoskeleton architecture and are activated by VEGF to control cell shape and motility [23]. RhoA and cdc42 are GGPP modified proteins whose function can be inhibited by lovastatin treatment [15]. Lovastatin induced dramatic changes in the actin cytoskeletal organization of HUVEC. Treatment with 0.5, 2 and 5 µM lovastatin for 24 hrs, resulted in a significant reduction of F-actin fibers stained with rhodamine-conjugated phalloidin and these fibers appeared disorganized (Figure 4A). In HUVEC and H28 MM cells, treatment with 0.5, 1 and 5 µM lovastatin for 24 hrs induced a dramatic up-regulation of both rhoA and cdc42 protein levels (Figure 4B). Cyclin D1 is a regulator of cell cycle progression and is up-regulated by a wide variety of cellular signaling pathways including rhoA activation [30]. The significant increase of rhoA protein levels did not result in up-regulation cyclinD1 protein levels but were reduced with lovastatin treatment of HUVEC and H28 cells (Figure 4B). Furthermore, employing a colorimetric rhoA activation assay, we determined the effect of lovastatin on VEGF_165_ induced rhoA activation in HUVEC and H28 cells. Serum starved cell extract represent inactive levels of rhoA while 0.2M GTP loaded extract represents fully active rhoA. As expected VEGF stimulation induced rhoA activity to approximately 60% of the GTP loaded activity. Lovastatin (10 µM, 24 hrs) inhibited VEGF_165_ induced rhoA activation in both HUVEC and H28 cells while co-administration of mevalonate (100 µM) and GGPP (10 µM) reversed the inhibitory effects of lovastatin (Figure 4C). These results demonstrate that lovastatin-induced rhoA is inactive likely due to the lack of GGPP modification.

### Inhibition of the VEGFR augments lovastatin-induced apoptosis

**Figure 5:**
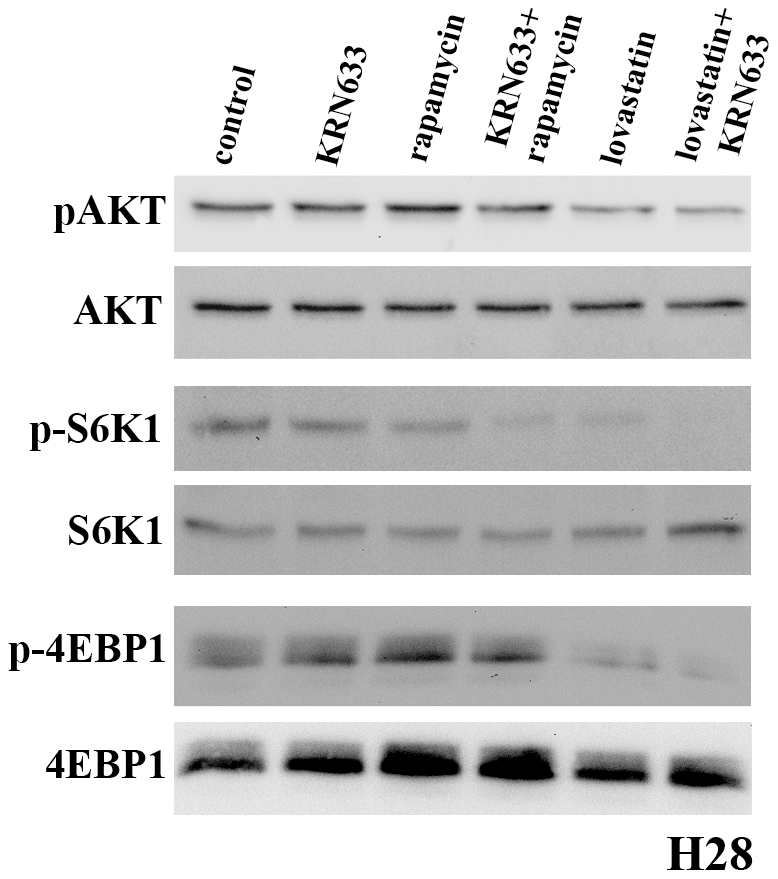
Lovastatin in combination with VEGFR-2 TKIs inhibits ligand induced activation of AKT, S6K1 and 4EBP1. Control cells were serum starved for 24 hr followed by 2 hr treatments with either 10 µM KRN633, 10 nM rapamycin, 5 µM lovastatin or their combinations. All cells were then lysed after stimulation with 50 ng/ml VEGF_165_ for 30 min. Results demonstrated that lovastatin in combination with KRN633 induced the most significant decrease in phosphorylation status of all three proteins in H28 cells. Expression levels of total AKT, S6K1 and 4EBP1 were assayed as loading controls.

Our previous studies have demonstrated that the combination of lovastatin and EGFR-TKI have resulted in synergistic cytotoxicity in a variety of human cancer derived cell lines [21]. Other studies have demonstrated the utility of combining EGFR-TKI with downstream inhibitors of the AKT pathway including rapamycin. Mammalian target of rapamycin (mTOR) plays a central role in regulating AKT driven translation initiation by regulating S6K1 and 4EBP1 activity [31]. Rapamycin has limited clinical activity due to a feedback loop that activates AKT and acquired resistance [31] suggesting that lovastatin may represent a novel therapeutic approach to target this pathway and enhance RTK-TKI activity. In this study, we evaluated the ability of rapamycin or lovastatin to augment the effects of the VEGFR-2 inhibitor KRN633. The H28 MM cell line had a relatively weak response to lovastatin-induced AKT inhibition. H28 cells express both VEGF and VEGFR-2. By Western blot analysis of activated AKT and its downstream targets S6K1 and 4EBP1, KRN633 and rapamycin treatments alone had minimal effects on the activation of these proteins. The combination of these agents showed enhanced inhibition of this pathway (Figure 5). In contrast, lovastatin treatment alone inhibited AKT, S6K1 and 4EPB1 phosphorylation and the combination of lovastatin and KRN633 induced a dramatic inhibition of the AKT pathway in this MM derived cell line (Figure 5).

**Figure 6:**
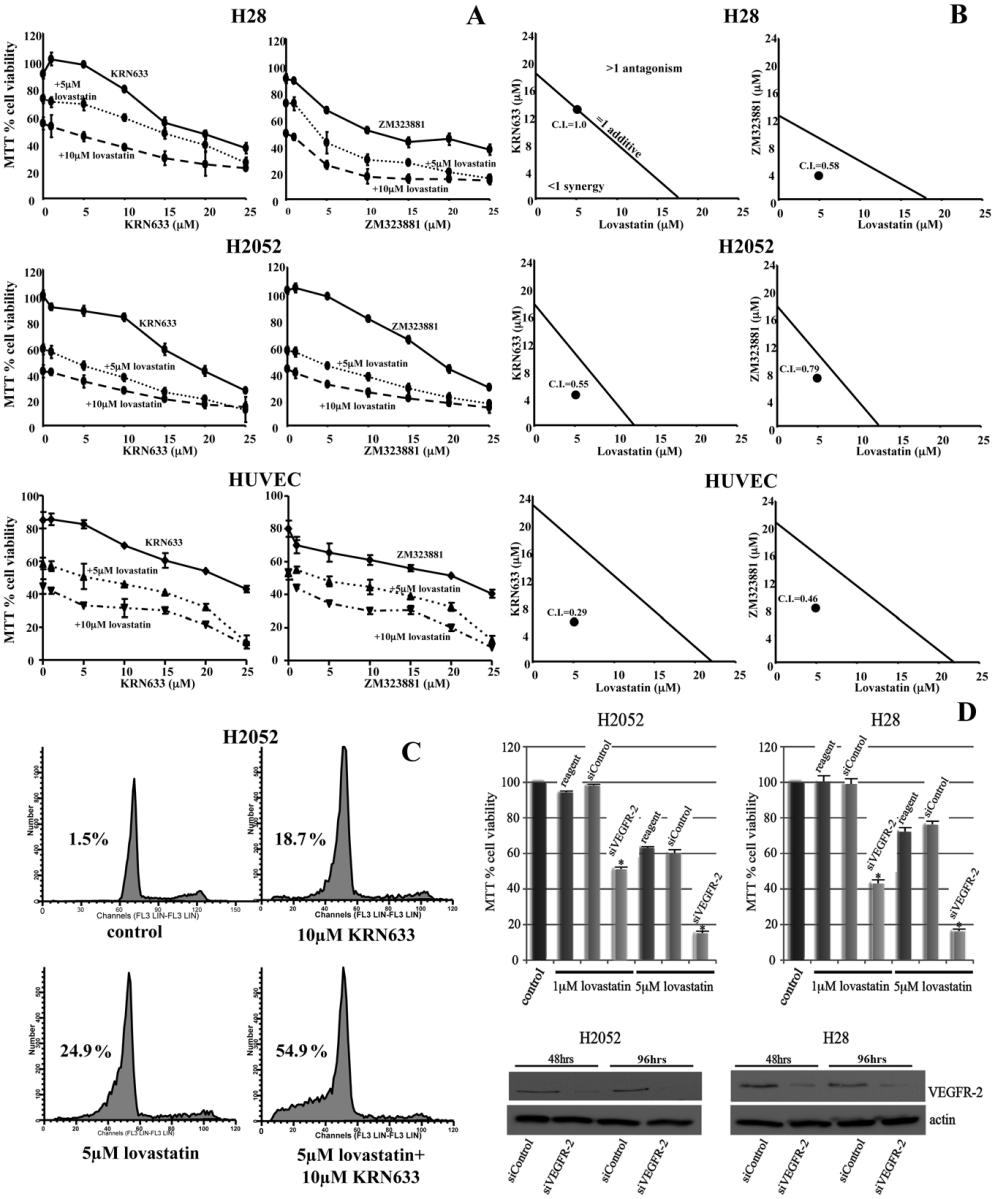
Combining lovastatin with VEGFR-2 TKIs induces synergistic cytotoxicity in MM cells and HUVEC. A, Evaluating the cytotoxic effects of treatment of lovastatin in combination with VEGFR-TKI on MM cell lines H28 and H2052 and HUVEC employing MTT Assays. The VEGFR-2-TKIs KRN633 and ZM323881 at doses of 1–25 µM were evaluated alone or in combination with 5 and 10 µM lovastatin. MTT data were normalized to untreated (media alone) cells (representing 100%) and is typical of 2 independent experiments. B, Isobologram analyses of the combination of 5 µM lovastatin and VEGF receptor inhibitor. MTT50 values were determined for 72 hr lovastatin and 48 hr VEGF receptor inhibitor treatments. MTT50 values are represented on the axes. The concentration of VEGF receptor inhibitor that demonstrated MTT50 with 5 µM lovastatin was plotted. Combination-Index (CI) was determined with CI<1, CI = 1, and CI>1 as synergism, additive effect, and antagonism, respectively [34]. C, Apoptosis was measured using flow cytometry analysis of H2052 MM cells following either control or combinations of 10 µM KRN633 with 5 µM lovastatin treatments. The percentage of apoptotic cells is shown in the upper left quadrant of each histogram. Results demonstrated that lovastatin in combination with KRN633 induced a potent apoptotic response in these cells. The data is typical of 2 independent experiments. D, H28 or H2052 cells were transiently transfected with 50 nM control (siControl) or VEGFR-2 (siVEGFR-2) siRNA oligonucleotides for 48 hrs. Cells were then treated with media or 1 and 5 µM lovastatin with fresh 50 nM siRNAs for an additional 48 hrs and analyzed for cell viability using the MTT assay. * P<0.001 comparing siControl to siVEGFR-2 in lovastatin treated cells as determined by paired T-test analysis. Total protein extracts in H2052 and H28 cells were analyzed by Western blotting for VEGFR-2 and actin following 48 and 72 hrs treatments with 50 nM of siControl and siVEGFR-2 siRNAs.

We further evaluated the combination of lovastatin and VEGFR-2 TKI on tumor cell cytotoxicity in HUVEC and MM cells. Utilizing MTT analysis and propidium iodide flow cytometry, we investigated the effects of combining two different VEGFR-TKIs with lovastatin on the viability of the H28 and H2052 MM derived cell lines and HUVEC. KRN633 inhibits VEGFR 1, 2 and 3 with similar kinetics while ZM323881 is highly selective for VEGFR-2 [32], [33]. With both MM derived cell lines and in HUVEC, increases in the concentration of the VEGFR-TKIs, KRN633 and ZM323881, resulted in a dose dependent decrease of MTT activity (Figure 6A). The pre-treatment of either 5 µM or 10 µM lovastatin for 24 hrs prior to the addition of 0–25 µM concentrations of the VEGFR-TKIs for 48 hrs resulted in co-operative cytotoxicity in both MM cell lines and HUVEC treated with either VEGFR-TKI (Figure 6A). The use of the Combination Index (CI) isobologram method of analysis [34] allowed for the determination of the effects of the combination of the lovastatin and VEGFR-TKIs (Figure 6B). CI values of <1, 1, and >1 are indicative of synergism, additive effect, and antagonism, respectively. The H28 MM cell line at the therapeutically relevant 5 µM dose of lovastatin resulted in a CI value of 0.58 for the combinatorial treatment of lovastatin and ZM323881, but the combination of lovastatin and KRN633 obtained a CI value of 1 (Figure 6B). The H2052 MM cell line and HUVEC had CI values of less than one for both VEGFR-TKIs. These results indicate that combining lovastatin with VEGFR-TKIs consistently induced synergistic cytotoxicity in MM and HUVEC cells.

To determine if this combination based approach resulted in enhanced apoptosis, we assessed MM cells treated with 5 µM or 10 µM of the VEGFR-TKIs alone or in combination with 5 µM lovastatin using the same experimental conditions as above. In both cell lines, with both VEGFR-TKIs tested, the combination with 5 µM lovastatin with 5 µM and 10 µM of the VEGFR-TKIs induced a more potent apoptotic response than either agent alone. Representative results for the H2052 cell line using the inhibitor KRN633 are shown (Figure 6C) and demonstrate a significant increase in apoptosis of the cells when the treatments were combined. Lovastatin treatment (5 µM) induced an apoptotic response that was significantly enhanced in combination with 10 µM KRN633 treatments (Figure 6C). Thus, the synergistic cytotoxicity observed with the combination of lovastatin and VEGFR-TKIs in MM cells is accompanied by a potent apoptotic response.

To further demonstrate the role of VEGFR-2 as a target of these VEGFR-TKIs in the synergistic cytotoxicity observed in combination with lovastatin in MM cells, we specifically targeted the expression of VEGFR-2 employing short inhibitory RNA sequences (siRNAs). Employing the MTT cell viability assay, we demonstrated that while the siControl treatments (50 nM 48 hrs, followed by 48 hrs lovastatin treatment) had no effect on lovastatin treatments (1 and 5 µM) compared to reagent alone, siVEGFR-2 (50 nM 48 hrs, followed by 48 hrs lovastatin treatment) significantly enhanced lovastatin-induced cytotoxicity in H2052 and H28 MM cells (Figure 6D). Western blot analysis confirmed the specificity of the siRNAs employed as siVEGFR-2 but not siControl targeted VEGFR-2 expression at 48 and 96 hr treatments (Figure 6D).

## Discussion

In our previous study, we demonstrated that the targeting of HMG-CoA reductase, which results in mevalonate depletion [16], can inhibit the function of the EGFR [21]. Furthermore, combining lovastatin with gefitinib, an EGFR-TKI, induced apoptotic and cytotoxic effects that were synergistic. This was demonstrated in several types of tumor cell lines and potentially involved the PI3K/AKT pathway [21]. The mechanisms regulating the inhibitory effects of lovastatin on EGFR function and the synergistic cytotoxicity in combination with gefitinib are currently not known. These findings suggest that mevalonate pathway inhibitors and receptor TKI may represent a novel combinational therapeutic approach in a variety of human cancers. The VEGFR and the EGFR are both members of RTK family that share similar activation, internalization and downstream signaling characteristics [3], [35]. Therefore, targeting the mevalonate pathway may have similar inhibitory effects on VEGFR and may also enhance the activity of VEGFR-TKI. VEGFR, particularly VEGFR-2, play important roles in regulating angiogenesis by promoting endothelial cell proliferation, survival and migration [7]. VEGF and VEGFR are also expressed by some tumor cells, like MM, acting in a functional autocrine loop capable of directly stimulating the growth and survival of MM cells [9].

In this study, we have shown lovastatin does indeed inhibit ligand-induced VEGFR-2 activation through inhibition of receptor internalization resulting in diminished AKT activation in HUVEC and MM cells. Lovastatin treatment re-organized the actin cytoskeleton, inhibited proliferation and induced apoptosis of HUVEC at therapeutically relevant doses (<5 µM) [29] despite addition of exogenous VEGF. AKT activation, which mediates cell survival, along with its downstream targets S6K1 and 4EBP1 were significantly inhibited by lovastatin treatment. Combining lovastatin with VEGFR-TKIs also induced synergistic cytotoxicity of HUVEC cells. Due to their role in promoting tumor neovascularization, inhibiting the function of VEGF and VEGFR has been the focus of a number of therapeutic approaches [1]. The limited clinical responses associated with these agents have been associated with their ability to promote disease stabilization and rarely induce tumor regression [1], [36]. Thus, agents that can co-operate and enhance the activity of VEGFR-TKI, like lovastatin, may increase their therapeutic activity.

MM is a highly aggressive tumor that is rarely curative and median survival is in the range of 10–17 months [11], therefore, novel therapies for MM are needed. Elevated levels of circulating and serousal VEGF in MM patients and the expression of VEGF and VEGFR on MM cells that can drive their proliferation and enhance their survival [9] has led to the evaluation of VEGFR targeted therapies. Bevacizumab, a monoclonal antibody against the VEGF, which is approved for the treatment of colon cancer, in combination with chemotherapy, failed to significantly affect outcome to chemotherapy treatment alone [37]. Various VEGFR-TKI employed a single agents also failed to demonstrate clinical utility in MM patients [37]. As like HUVEC, MM cells also depend on VEGFR signaling, we also examined the effect of lovastatin alone and in combination with VEGFR-2 TKI on MM cell viability. Combining 5 µM lovastatin treatments with two VEGFR-2 inhibitors in the H28 and H2052 mesothelioma derived cell lines demonstrated synergistic cytotoxicity through the induction of a potent apoptotic response. These results highlight a novel mechanism regulating VEGFR-2 function and a potential novel therapeutic approach for MM.

Inhibition of HMG-CoA reductase has been evaluated as an anti-cancer therapeutic approach owing to its ability to inhibit tumor cell proliferation, induce tumor specific apoptosis and inhibit cell motility and metastasis in several tumor models [38]–[41]. A number of Phase I Clinical trials evaluating the efficacy of high doses of lovastatin failed to demonstrate significant anti-tumor activity [29]. The tumor types evaluated in these studies did not include those that we identified as being highly sensitive to lovastatin-induced apoptosis, including head and neck squamous cell carcinomas and cervical carcinomas [39]. As a result, a Phase I clinical evaluation of lovastatin in recurrent head and neck squamous cell carcinomas and cervical carcinoma patients was undertaken by our group. Although no tumor regressions were observed, 23% of patients exhibited stable disease [28]. Taken together, the most effective use of lovastatin and VEGFR-TKI would be as part of a combined modality approach.

Due to the potential for mevalonate metabolite depletion to functionally alter the VEGFR signaling pathway, HMG-CoA reductase and VEGFR targeted therapies may be associated. This study has shown that the combination of lovastatin with two VEGFR-TKIs induced significant co-operative cytotoxicity in both MM cell lines tested. More detailed isobologram analysis demonstrated that this enhanced cytotoxic response was synergistic. These results suggest the potential of combining these two therapeutic approaches. The inhibition of mevalonate synthesis and the depletion of one or more mevalonate metabolites is the mechanism regulating this phenomenon. The combination of statins and VEGFR-TKI represents an attractive therapeutic approach as clinical trials have shown a different spectrum of toxicities with these agents [29], [36]. In a recent manuscript, we have demonstrated similar inhibition of EGFR function by lovastatin in squamous cell carcinoma cells [42]. While in vivo murine tumor models evaluating the efficacy of statins have been employed, differences in drug metabolism between species and lack of target validation in many studies suggests the potential of off target effects playing a role in statin response [43], [44], [45]. To circumvent these issues, we evaluated the BR.21 NCIC-CTG Phase III clinical trial of the EGFR-TKI inhibitor tarceva as a single agent in non-small cell lung carcinoma patients [46]. In this trial, patients on erlotinib that were also taking statins to treat hypercholesterolemia had a trend to better outcomes than patients on erlotinib alone [42]. These studies have led to a Phase I/II clinical trial at our institute combining cerivastatin and erlotinib that is currently accruing patients (ClinicalTrials.gov Identifier**:** NCT00966472). Similar data for statin usage in VEGFR-TKI treated MM patients were not available due to the lack of a sufficient patient population for analysis. The ability of lovastatin to inhibit both EGFR and VEGFR function is intriguing and requires further study to elucidate its underlying mechanism. This suggests the potential for HMG-CoA reductase inhibition to affect the activity of a number of RTK potentially through a similar, novel and as yet uncharacterized mechanism.

### Ethical Statement

Not applicable with respect to this study.

## Materials and Methods

### Tissue Culture

Human Umbilical Vein Endothelial Cells (HUVEC) (Clonetics, lot 2F1276, Walkersville, MD) were maintained in EGM-2 media supplemented with 2% fetal bovine serum provided in the EGM-2 Single Quot Kit Supplements and Growth Factors (Lonza, East Rutherford, NJ). The human mesothelioma lines, NCI-H28 and NCI-H2052, were obtained from the American Type Culture Collection (ATCC, Rockville, MD) and maintained in HyQ DMEM/High Glucose (HyClone, Logan, Utah) supplemented with 10% fetal bovine serum (Medicorp, Montreal, QC). The cell lines used in this study were exposed to solvent control or lovastatin (provided by Apotex, Mississauga, ON; diluted from a 10mmol/L stock in ethanol), or human recombinant VEGF_165_ (provided by National Cancer Institute, Rockville, MD; reconstituted to a 50 mg/ml stock in deionized water) at a concentration of 50 ng/ml. The mesothelioma cell lines were exposed to solvent control or VEGFR-2 Inhibitor V, ZM323881, or VEGFR-TKI III, KRN633 (Calbiochem; both reconstituted to a 1mmol/L stock in DMSO). The siRNA oligonucleotides used in this study were purchased from Dharmacon (Boulder, CO). siControl: siGENOME non-targeting siRNA, siVEGFR-2: siGENOME SMARTpool human KDR. Transfection procedures were performed with DharmaFECT-4 reagent (Dharmacon) in both H28 and H2052 cells according to the manufacturer's protocols. Cells were grown on 6-well plates or 96-well plates and transfected with 50 nM of the siRNAs. After two days incubation, cells were treated with media or 10 µM lovastatin for another 48 hrs. The cytotoxic effects of lovastatin remained consistent in all three cell lines throughout the course of these experiments.

### 3-(4,5-Dimethylthiazol-2-yl)-2,5-Diphenyltetrazolium Bromide Assay (MTT Assay)

In a 96-well, flat-bottomed plate (Fisher, Mississauga, ON), ∼7500 cells/150 µl of cell suspension were used to seed each well. The cells were incubated overnight to allow for cell attachment and recovery. Following a 48- or 72-hr treatment of lovastatin, ZM323881, KRN633, or a combination of lovastatin and a VEGFR-TKI, 42 µL of a 5 mg/ml solution in PBS of the MTT substrate (Sigma) was added and incubated for up to one hr at 37°C. The resulting blue-brown formazan precipitate formed was solubilized by the addition of 84 µL of a 0.01M HCl/10%SDS (Sigma) solution and incubated for 8 hrs at 37°C. The plates were then analyzed on a Dynex Technologies MRX Microplate Reader at 570 nm using the Revel software (Dynex Technologies, Chantilly, VA) to determine the absorbance of the samples. Treatments were performed in replicates of six and the means expressed as the percent viability relative to the untreated control (100% viable). Statistical analysis: Combination Index (C.I.) was determined by the method of Chou and Talalay as previously described [34]. P values were determined by standard paired T-test evaluations.

### Cell Viability Assay

In a 6-well flat-bottomed plate (Fisher), ∼500000 HUVEC were used to seed each well. The cells were incubated overnight to allow for cell attachment and recovery. Following 72 hr treatment using solvent control or lovastatin in the presence or absence of 50 ng/ml VEGF_165,_ the cells were trypsinized and collected. The number of viable cells in 500 µl of each sample was subsequently counted on the Beckman Coulter Vi-Cell-XR Cell Viability Analyzer (Mississauga, ON). Treatments were performed in triplicates. Data were normalized to the untreated control.

### Propidium Iodide Flow Cytometry

In 10-cm plates (Fisher), ∼3.5×10^5^ mesothelioma cells or ∼5×10^5^ HUVEC were used to seed each plate. The plates were incubated overnight to allow for cell attachment and recovery. The HUVEC were treated with solvent control or lovastatin, in the presence or absence of 50 ng/ml VEGF_165_ for 72 hrs. The mesothelioma cells were treated with solvent control or lovastatin. Following a 24-hour pre-treatment with lovastatin alone, solvent control or VEGFR Inhibitor (KRN633 or ZM323881) was added for an additional 48 hrs. After the desired treatment length, the media, PBS wash and trypsinized cells were collected in the same 50 mL conical tube. The collected cells were fixed with 80% ethanol and incubated at −20°C for a minimum of 24 hrs. The cells were washed once then resuspended in staining buffer containing 50 µg/ml propidium iodide (Sigma) and 100 µg/ml RNaseA (Invitrogen, Carlsbad, CA). Ten thousand cells were evaluated using the Beckman Coulter Epics XL Flow Cytometer and the percentage of cells in pre-G_1_ phase was determined using the ModFit LT program (Verity Software House, Topsham, ME).

### Western Blot Analysis

Total cellular protein was extracted using a buffer that consisted of 50 mM Tris-HCl pH 7.5, 150 mM NaCl, 0.25% sodium deoxycholate (Sigma), 1% IgePal, 0.1% SDS (Sigma), 1 mM EDTA, 5 mM sodium fluoride (Sigma), 1 mM sodium orthovanadate (Sigma), and protease inhibitor cocktail (Sigma; diluted from a 10× stock). Approximately 100 µL of extraction buffer was used per plate. Total protein was quantified with the BCA Protein Assay Reagents (Pierce, Nepean, ON) using bovine serum albumin (Sigma) for the standard. Protein extracts representing 50 to 100 µg total protein were separated on SDS-PAGE gel using the BioRad Mini Protean 3 System (Bio-Rad Laboratories, Hercules, CA) and electro-blotted onto Hybond P PVDF membranes (Amersham, Piscataway, NJ). Membranes were blocked in 5% skim milk powder in PBS/0.02% Tween (Sigma) for an hour at room temperature. Primary antibody, diluted in 5% skim milk powder in PBST, was incubated with the membrane overnight at 4°C. The primary antibodies used were specific for VEGFR-2, RhoA, cdc42, cyclinD1 (Santa Cruz Biotechnologies, Santa Cruz, CA); phospho-AKT, AKT, phospho-S6K1, S6K1, phospho-4EBP1, 4EBP1 (Cell Signaling Technology, Danvers, MA); and actin (Sigma). The peroxidase-conjugated AffiniPure Goat Anti-mouse/rabbit IgG (Jackson ImmunoResearch, West Grove, PA) secondary antibodies were applied at a 1∶5000 dilution and the peroxidase-labeled Affinity Purified Antibody to goat IgG (KPL) secondary antibody was applied at a 1∶1000 dilution in 5% skim milk powder in PBST and incubated for a minimum of an hour at room temperature then processed for detection with the Supersignal West Pico Chemiluminescent Substrate (Pierce), using the Gene GNOME Imager and Genesnap Imaging Software (Syngene, Frederick, MD). After the desired exposure was obtained, the membrane was stained with Ponceau Red (Fisher) to ensure equal loading of the samples. Membranes were stripped using Restore Western Stripping Buffer (Pierce, Nepean, ON) to allow for a second probing.

### Pinpoint Cell Surface Protein Labeling

The Pinpoint Cell Surface Protein Isolation Kit (Pierce) was used to identify and isolate cell surface proteins following the manufacturer's instructions. In brief, control or 24 hrs lovastatin treated HUVEC cells were stimulated with or without 50 ng/ml of VEGF for 30 min. Cells were then washed with ice-cold PBS and surface proteins were biotinylated and isolated using immobilized avidin, prior to Western blot analysis of VEGFR-2 and actin levels as described above.

### Phalloidin Staining/Immunofluorescence

In a 6-well flat-bottomed plate (Fisher), glass cover slips (Fisher) were placed into each well and ∼250000 cells were used to seed each well. The cells were incubated overnight to allow for cell attachment and recovery. Following a 24 hr treatment of solvent control or lovastatin in serum-free media, the HUVEC cells were treated with recombinant humanVEGF_165_ for 30 min prior to fixation. The cells were subsequently washed with PBS then fixed with 4% paraformaldehyde (Sigma) buffered in PBS for 15 min at 37°C and stored in PBS at 4°C. To visualize actin cytoskeletal architecture, 100 µL of a 1 ng phalloidin-rhodamine conjugate in PBS was used to treat each cover slip containing the attached HUVEC cells for 15 min in the dark. Prior to immunofluorescence staining, the cells were permeabilized with PBS+0.2%Triton X-100 (Sigma) for 15 min. The cells were blocked for 30 min with PBS+3%FBS then incubated with the VEGFR-2 antibody at a dilution of 1∶50 in PBS+3%FBS for an hr. The cells were then blocked with PBS+5% chicken serum (Sigma) for 30 min. Following the second blocking, the cells were incubated with Alexa Fluor 488 chicken anti-mouse IgG (Molecular Probes, Carlsbad, CA) at a working dilution of 10 µg/ml in the dark for an hr. The cells were then mounted to a microslide with DAPI mounting media (Vector Laboratories, Burlingame, CA) and analyzed under fluorescent microscopy using the Axiovision software (Allied High Tech Products, Rancho Dominguez, CA).

### Rho A Activation Assay

The HUVEC and H28 cell lines were cultured in serum free media treated with 10 µM lovastatin for 24 hrs with or without 100 µM mevalonate or 10 µM GGPP. Cells were stimulated with 50 ng/ml EGF for 30 min to activate rhoA. Cell lysates were either snap frozen and stored in liquid nitrogen or used directly with the RhoA G-LISA kit (Cytoskeleton, Denver, Co) according to the manufacturer's instructions. This assay is based on the principle that a Rho-GTP-binding protein is linked to the 96-well plates. The active GTP-bound Rho in the cell lysates binds to the wells, while the inactive GDP-bound Rho is removed during the washing steps. The bound active RhoA is detected with a RhoA specific antibody and quantified by absorbance. The degree of RhoA activation is determined by comparing readings from the activated cell lysates (addition of 0.2 mM GTP) versus the non-activated cell lysates (serum starved cultures).
